# The influence and mechanism of health expenditures on investment of financial assets decisions: A case study of China's economy

**DOI:** 10.3389/fpubh.2022.994620

**Published:** 2022-11-10

**Authors:** Zhanqiang Shao, Lingling Dou

**Affiliations:** ^1^School of Finance, Nankai University, Tianjin, China; ^2^School of Statistics and Big Data, Henan University of Economics and Law, Henan, China

**Keywords:** health expenditures, non-financial assets, capital formation, dynamic ARDL approach, China

## Abstract

Policymakers worldwide have been actively involved in the past few decades to ensure that human diseases are kept to a minimum. A new econometric technique, dynamic ARDL simulations, was used in this study to estimate and model the influence of health expenditures on investment in non-financial assets in China from 1990 to 2019. An economic growth framework, gross capital formation, information and communication technologies, foreign direct investment, and carbon emissions are all considered in the empirical model—the analysis produced interesting results. First, the estimates show that health expenditures and foreign direct investment have a significant long-run decreasing impact on non-financial assets in China by 0.451 and 0.234%. Second, economic growth and gross capital formation significantly affect the economy's non-financial assets. Likewise, ICT and carbon emissions also positively correlate with an explained variable in China. The findings show that the economy is becoming less investment-intensive as health spending and foreign direct investment rise. The study develops important policy implications for the selected country to achieve desired targets based on the empirical results.

## Introduction

The rising cost of health care services is quickly becoming a major concern for governments worldwide. The majority of growing economies and international health care spending growth are increasingly occurring outside the targeted economies' borders. Countries like the People's Republic of China see rapid increases in health care spending (hereinafter referred to as China). Chronic diseases are a major factor in healthcare costs in developed and developing countries. The fast-growing emerging economies are, however, saddled with a double whammy. Despite efforts to eradicate infectious diseases, these economies are also grappling with an increase in the prevalence of no communicable diseases (NCDs) (1).

However, the transaction sector also needs investments in fixed assets, whose state determines the effectiveness of transactions (the value of transaction costs). There may be a financial crisis resulting from the deflation of the housing bubble, the failure of the banking structure, and the mounting debt (2). Secondly, long-term socio-economic issues, such as rising unemployment, expanding discrimination, austerity programs, welfare cuts, and limited industrial effectiveness in a globalized world, will significantly impact in the next 5–10 years. Environmental issues such as environmental change, biodiversity loss, ocean acidification, chemical contamination, freshwater, and land use issues are all transgressing planetary boundaries in the long term (10–50 years). A new scientific study stream has formed in response to these environmental issues. It argues that large-scale sustainability transitions in transportation, energy, and agri-food socio-technical systems are required to address them (3). The multi-level perspective in this research stream hypothesizes sustainability alterations as green niche innovations battling against existing (unsound) systems and possibly substituting or reconfiguring these systems [e.g., (4–6)]. Health problems are on the rise as a result of an unsustainable environment (7).

In many affluent countries, reforming health care delivery systems is a continuous effort. Increasing or decreasing public funding health care spending is one of the simplest ways governments can adjust their health care delivery systems. The idea of a more significant role for the public in health care has been floated around the world regularly—others around the globe urge for a diminished public role (8). As a result, it's critical to understand the full ramifications of any changes to the health care system that alter the proportion of public funding. In the end, what matters is the impact on health outcomes of such modifications. Financing medical expenses using tax dollars can help people get the necessary care. Increasing public funding, on the other hand, may result in less efficient healthcare delivery and worse health outcomes. There has been relatively little attention paid to this empirical subject until now. The extent to which health care is publicly financed varies considerably among nations and over time. According to similar economies (9), China has a relatively low level of public financing. However, in other countries, such as the United States, it has risen from 24% in 1960 to 41% in 1985. The growth in Canada has been the same amount, but the public share in Canada has been higher than in the United States. Other countries' public funding shares have remained generally stable but varying amounts. As of 1960 and 1993, public funding in the United Kingdom was 85 and 86%, respectively. Health outcomes vary between countries for a variety of reasons, not only the amount of money spent on public health care (6). Aside from socio-economic variables, demographics, and health behaviors, these factors may also have a role. Factors peculiar to a given country may also impact the health of its citizens. A comprehensive analysis of the factors influencing results in different nations and across time is required. Preliminary research has focused primarily on health issues (3).

Financial institutions are interested in the tougher definition of “reputation” that requires recurrent interactions between economic actors. There is a lack of empirical data to support the importance of reputation building in the capital markets. Even though banks keep meticulous records of the loans they provide and the repayments they receive, they rarely divulge information about the unsuccessful customers they've turned away. As a result, new approaches to research are needed. For example, Mujasi et al. (10) use a panel of banknote data to study how a funding institution might benefit from its reputation and serve the health sector.

The Information & Communication Technology for countries across the globe significantly contributes to health facilities and financial investment. Its importance is that it provides significant financial incentives and penalties to healthcare institutions. Increasingly relevant are questions about the financial and productive benefits of widespread usage of information and communication technology (ICT) (8). There are many benefits to using ICTs in healthcare, such as improving the quality of care, but they can also harm an organization's financial performance and productivity (11, 12). Hospitals that can prove they are using modern technology in a meaningful way are rewarded by the Centers for Medicare & Medicaid Services (CMS). In order to increase safety, quality, and competence, decrease health inequalities, engage patients and families in treatment decisions, improve public health, and ensure the privacy and security of persistent health information, the meaningful use effort supports many health care goals. To enhance clinical after-effects, population health outcomes, transparency and efficiency, authorized individuals, and more robust associations with non-financial assets are expected consequences of meaningful use of compliance (13).

Few empirical research has centered on the impact of health expenditures on financial investment decisions, even if many have looked into the impact of health expenditures on other economic variables such as income disparity, environmental quality, energy investment, etc. Spending on healthcare often necessitates reprioritizing other family expenses (14). It can pose a danger to living standards, which can have immediate and long-term effects on a family's financial security. Therefore, the case study of 10 Asian nations, including low-income countries, revealed a high incidence of such expenditure in South Korea when considering Korea's GDP per capita (15). An analysis of 59 nations by Hu et al. (16) found that South Korea had a higher-than-average % of households experiencing catastrophic expenditures, defined as spending more than twice their income on non-food items. According to a study looking at the development of catastrophic health expenditures in Korea, the %age of households experiencing catastrophic expenditures has been growing steadily over the past few years, peaking at 2.15% in urban areas and 3.44% in rural regions in 2005 (17).

Health care costs are likely to arise frequently in China, as evidenced by the aforementioned local and international findings. As a result, China's economy may be at risk of suffering from health problems, especially if remedial measures such as healthcare spending are necessary. Health spending and financial investment were broken out and contrasted in this study. Health spending was also analyzed in relation to financial investment and a number of other variables of interest in China. The results reveal the potential effects of health expenditures on financial investment. However, the impact assessment is limited due to the use of time series data. Since no Chinese study has yet attempted to answer the question of whether or not rising healthcare costs will lead to a shift in financial asset allocation, this remains the country's biggest obstacle. This is the central issue and the focus of our investigation into the Chinese economy.

This study has the following contribution to existing literature. The first contribution of this study is to investigate the connection of health expenditures with investment in non-financial assets. The financial-economic crisis and health expenditures have a deeper cultural problem in modern capitalist societies. According to this theory, a new historical age has dawned on modern cultures. It is characterized by a convergence of financial, socio-economic, and health-related issues. Therefore, over time there is an increasing trend in health expenditures which may cause a decline in financial investment. Thus, it is necessary to investigate the role of health expenditures for financial investment in China's economy (18). The second objective is to investigate long-wave dynamics for economic growth, foreign direct investment, ICT, and environmental quality. Those who hold this view rely on a foundation of evidence. Economic and social downturns are a regular and inevitable aspect of major transitions. A move from the “installation period” of the ICT paradigm to the “deployment phase” is seen in the light of the current state of ICTs as a signal for financial discipline and a refocus on long-run investments in the real economy (11). The merging of the ICT paradigm and other indicators of financial investment may provide a system to overcome the current crisis. A third view emphasizes less the advanced econometric techniques investigate the long-run association among variables. Therefore, this study used the advanced form of econometric approach in which Dynamic ARDL and advanced causality test promises to solve serious problems. Some argue that financial investment and other issues compete for political and societal attention because they have varying timeframes, causes, and solutions. Investors' confidence may be eroded due to shifting social and political objectives.

## Literature review

The study of the factors that may influence a person's financial wealth has been undertaken numerous times over the years. Numerous country-specific studies of the bi-variate framework are available in the academic community's collective brain trust (19). The confluence of numerous financial variables has mostly gone unnoticed or has been overlooked in panel research (20, 21), however. Inconsistent results have been achieved due to elements like these being overlooked, and these conclusions are of little use to policymakers in terms of financial development or policy implications. The various variables must be connected in some way (22).

Studies on health and related issues with a wide range of variables are summarized in this study. It is believed that health indicators, economic growth, and the environment are linked. The health effects of rising CO_2_ emissions are seen worldwide, but they are most acute in developing countries. According to Persyn and Westerlund (23), an increase in health expenditure magnified both economic growth and CO_2_ emissions in the OECD countries. Timilsina (24) and Wu et al. (25) conducted a similar study in the Middle East and North Africa (MENA) region and found that health expenditure is a source of increasing economic activity at the expense of the environment. Shahzad et al. (26) found that health expenditure is the driving indicator for CO_2_ emissions in 20 countries. Hao et al. (27) and Song et al. (28) investigated the link between environmental pollution and health effects in China along similar lines and found that the environment is likely to cause major health concerns. Yunfeng and Laike (29) have taken a closer look at the impact of power generation and health expenditure on the environment in Caribbean countries. They maintained that rising healthcare costs contributed to the environment's degradation. It was found by Cui et al. (30) in a study of panel data from 94 nations that government expenditure has a direct moderating effect on pollution and promotes economic growth; they did not find any negative impact on health (31). Health expenditures, economic growth, and the environment have been shown to correlate positively in several empirical types of research (32–34). However, some researchers, such as Dong et al. (35), Du and Li (36), Rao et al. (37), and Wu et al. (38), reject these conclusions, claiming that health expenditures have benefitted the economy and the environment.

In the last few years, there has been a heightened interest in figuring out the impact of financial inclusion on health care costs, health conditions, and wellbeing (39). Over one billion adults have approached an account since 2011, according to The World Bank (40), and considerable strides are being made globally to involve greater people in financial services of their critical role. More than 69% of the world's adults now have a bank account, yet the latest Fundex statistics show that 31% of adults are still unbanked (1.7 billion people, or 31%) (41). Most of those without bank accounts are women and members of low-income families living in rural areas. Since financial inclusion is critical to the seven SDGs, countries agreed to work together globally and commit themselves to China and other countries to develop high-Level Ethics for Digital Financial Inclusion (40). Some nations use more than 12% of GDP on health care, while others use < 3% of GDP on it. As a result, it is clear that as nations go up the financial ladder, their health expenditure differs substantially from those at the bottom of the scale. This is the case with China, categorized as an upper-middle-income nation by the World Bank. Differences in growth and development have resulted in a significant gap in health costs around the globe. In this part, we examine various relevant researches at the individual and cross-country levels, which have examined the link between financial inclusion and health spending (42, 43).

The results are consistent no matter how many different estimation methods are used. Zhang et al. (44) used the 2SLS technique of estimation and Lewbel's instrumental variable approach to examine the influence of financial inclusion on domestic health expenses in Ghana. According to the findings, households' out-of-pocket health care expenses rise by 0.1367–1.7608 for every unit increase in financial inclusion. Women-headed households and those in metropolitan regions are more likely to experience these consequences. One of their findings was that financial inclusion captures higher expenditures on medical items than casualty care. Similarly, research by Li et al. (45) examined the link between financial development and health expenditures in SSA countries. Finances have a favorable and significant impact on health care spending, according to the 2SLS estimating approach. This finding was supported by a study examining the effect of financial development on private health care expenditure. According to the results, an increase in bank branches per 100,000 people of 10% correlates to a 1.1% rise in private health care spending as a %age of GDP. An increase of 1.7 and 1% in the share of private health care spending is also attributed to broad money and bank credit available to the private sector as a %age of GDP. It was discovered in another study by Cao et al. (46), using Quantile Treatment Effects (QTE) to capture the effect of household credit on education and health spending by the poor in Vietnam. The influence of formal and informal credit on health expenditures was evaluated, as well as any differences in their impact on health expenditures. Informal credit had a favorable influence on health expenditure, but only at a very small level, while formal credit had a doubled effect. As a result, credit considerably impacts the health and education spending of the poor in Vietnam's peri-urban areas. Individuals' financial and overall asset holdings rise when they have access to savings accounts, as Chang and Han (47) discovered field trial in Nepal. As a result, their income rises as well. They conducted a field experiment to see if having access to a bank account affects household spending. The findings show that access to financial services positively impacts health care costs. Before the conclusion of her field trial, to acquire more reliable results, she analyzed the complete population that had just had a health crisis and revealed a considerably stronger statistically significant influence. Because of this, it was found that families spend more on care in the early stages of a health problem to avoid long-term costs. Thanh and Duong (48) study on health shocks and microcredit in rural areas of Vietnam found inconsistent results regarding the role of microcredit in alleviating health shocks, which mostly depended on the type of health distress. Health shocks tend to be better handled by households with access to microcredit than households without access to micro-credit. Zhong et al. (6) investigated the long-run linkages between economic and financial risk in the MINT economies from 1984 to 2014. They employed the Wavelet analysis and found the one-way causal association from economic risk to financial risk for Mexico and financial risk to economic risk for Turkey. Another case study in Germany (9) investigated the impact of the stock market index from 1996 to 2020. They employed the Markov Switching Regression and pointed out that the pandemic uncertainty index and real effective exchange rate disturb the German Stock Market index. Bilger and Manning (49) investigated the impact of financial development on MINT economies' carbon emissions from 1969 to 2019. They used the Rolling-Window Bootstrap estimation and showed the feedback hypothesis between the financial development and carbon emissions in selected economies. The study of South Korea related to exchange rate and economic progress from 1997 to 2021 by (50) and validated the positive relationship between economic progress and exchange rate. Another case study of China by Moura et al. (7) tried to investigate the impact of the economic complexity index on environmental quality from 1985 to 2019. Using quintile on quintile regression showed the positive impact of economic complexity on environmental quality.

In conclusion, prior research did not fully account for their crucial role in impacting environmental quality when selecting supply-side clean and green energy. The study's primary objective is to quantify the global scope of the growing issue, focusing on emerging economies. Consequently, the research has filled this need by focusing on the production side of green energy that may lead to environmental issues. In light of these restrictions, we employ a visually appealing panel model to investigate the impact of different forms of renewable energy generation—including nuclear, hydro, wind, and geothermal—on environmental degradation. The results shed fresh light on the topic, making this study an important addition to the existing empirical and theoretical work. Furthermore, this study contributes to the literature by delving into the effect of economic development and R&D expenditure on environmental quality in a subset of economies (here, the E5 economies) from 1990 to 2019. Moreover, the results for Generalized Dynamic Method of Moments (GMM) and Fully Modified Ordinary Least Square (FMOLS) will provide appropriate policy ideas for selected economies to achieve low environmental degradation and deal with the production side difficulties and desired environmental goals.

## Data and methods

### Theoretical framework

In this research, the estimation of health outcomes is based on a framework provided by Amuedo-Dorantes and Pozo (51). The level of investment in health is a choice individuals make in his model to maximize benefit throughout a lifetime. Grossman defines health expenditures as a combination of medical care, time spent on health, and education as a technology shifter. He does concede that other market products, such as housing, diet, recreation, cigarette smoking, and alcohol use, could be in the production function together with medical treatment (4). Using Grossman's and others' research and the studies discussed above, the current work specifies health production functions across countries and over time. In the United States, for instance (5) construct mortality regressions across states with factors such as per capita healthcare spending, per capita income, per capita cigarette usage, and per capita education. With microdata for the United States from 1959 to 1962, Bukari et al. (8) estimate a health production function using morbidity measurements. Using a battery of physiological indicators like blood pressure and cholesterol, the researchers find just a marginal impact of medical treatment on patient outcomes. Among the indicators of health used by Yang et al. (3) are a person's blood pressure, functional restrictions, and the person's health assessment. They define health in terms of educational attainment and other demographic factors. While education and health literacy are independent variables in Mujasi et al. (10) health production function estimations, health behavior is the dependent variable. Health (H) is defined here as a function of health care inputs (HC), health-related behaviors (HB), and other tradable (X). Furthermore, the DEM of a population has a direct impact on health outcomes due to innate biological mechanisms.

**Figure 1:**
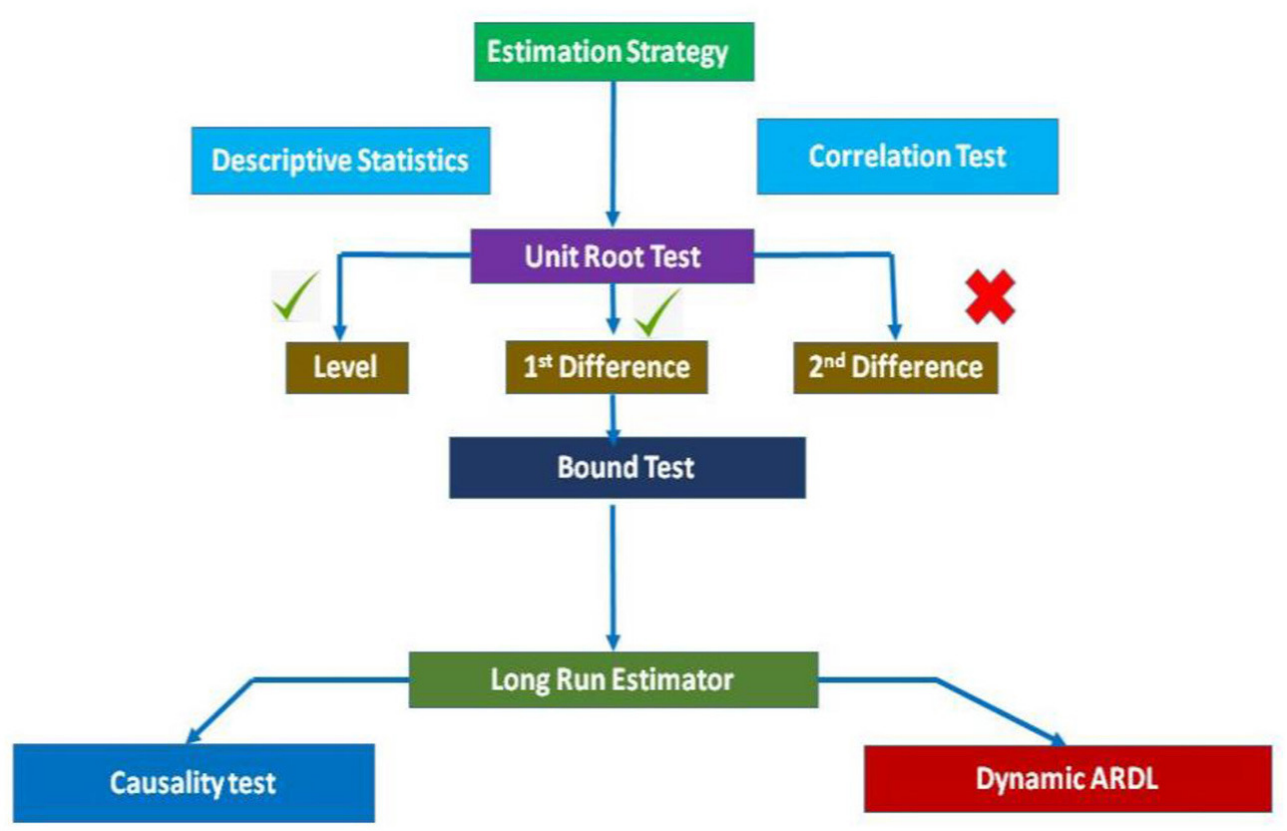
Estimation strategy flow chart.

Moreover, the estimation strategy is given as follows in Figure 1.

This research inspected the impact of health expenditures and economic growth with other variables on investment for non-financial assets in china by applying annual time series data from 1990 to 2019 obtained from WDI (40). Net investment in non-financial assets (NFA) were measured as % of economic growth. Economic growth was measured in US current $, the gross capital formation was measured by % of GDP, information and communication technology was measured by % of the population, foreign direct investment was measured by % of GDP, carbon emissions were measured in Kt, and the data relevant to such factors were collected from the World Development Indicators (WDI). All series were converted to logarithmic form to remove the issue of heteroscedasticity. The dynamic autoregressive distributed lag simulation methods were utilized in this study to analyze the actual change in the dependent variable induced by the independent variable (52, 53). We must first do a unit root test to verify the stationarity of each variable and the order of integration of the relevant variables before proceeding with dynamic ARDL simulations. There's a chance that non-stationary data will produce erroneous regression conclusions. Each variable was tested for stationarity at the level and the first difference. Non-stationary variables at the level term will have unit roots; if the first differences of the time series are stationary, this indicates that they are integrated of degree one or I (1). In order to run dynamic ARDL simulations, you must only employ stationary variables at either I(0) or I(I). These tests and enhanced versions of Dickey-Fuller, Phillips-Peron, and the Kwiatkowski-Phillips-Schmidt-Shin (KPSS) unit root tests were used to examine the series' linear stochastic trend. The following general equation is given to evaluate the relationship between the variables under investigation:


(1) $$\begin{array}{l} LNFA_{\text{t}}=\beta_{0}+\beta_{1}LHE_{\text{t}}+\beta_{2}LGDP_{\text{t}}+\beta_{3}LGCF_{\text{t}}+\beta_{4}LFDI_{\text{t}}\\ +\beta_{5}LICT_{\text{i}}+\beta_{6}LCE_{\text{t}}+u_{\text{t}} \end{array}$$

In the overhead Equation (1), β_0_ is constant, 1–6, β values are the coefficients of independent variables, and ε_*t*_ is the error term.

### Autoregressive distributed lag bounds test

The long-term connection between the research variables was examined using a bound test. The following ARDL bound test model was used to investigate the long-term association between the variables of interest based on the hypothesis:


(2) $$\begin{array}{l} \Delta LNFA_{\text{t}}=\delta_{0}+\delta_{1}LNFA_{\text{t}-1}+\delta_{2}LGDP_{\text{t}-1}+\delta_{3}LGCF_{\text{t}-1}\\ +\delta_{4}LFDI_{\text{t}-1}+\delta_{5}LICT_{\text{t}-1}+\delta_{6}LCE_{\text{t}-1}\\ +\overset{q}{\underset{i=1}{\sum }}\beta 1\;LHEt\;+\overset{q}{\underset{i=1}{\sum }}\beta 2\;LGDPt\;+\overset{q}{\underset{i=1}{\sum }}\beta 3\;LGCFt\;\\ +\overset{q}{\underset{i=1}{\sum }}\beta 4\;LFDIt\;+\overset{q}{\underset{i=1}{\sum }}\beta 5\;LICTi\;+\overset{q}{\underset{i=1}{\sum }}\beta 6\;LCEt\;+u_{\text{t}} \end{array}$$

Equation (2) shows the first difference, LNFA is the natural log of non-financial assets, LGDP the economic growth, LGCF gross capital formation, LFDI the log of foreign direct investment, LICT is the log of information and communication technology, and LCE is the natural log of carbon emissions, and t-i represents the optimal selection of lags according to the Akaike information criterion. To test for long-term correlations between the research variables, and will be studied. We estimate both the short- and long-term ARDL models because of long-term connections between the research variables. The following are the null and alternative hypotheses for the bound test:


$$\begin{array}{lll} H_{0} & = & \delta_{1}=\delta_{2}=\delta_{3}=\delta_{4}=\delta_{5}=\delta_{6}\;=\;0\\ H_{1} & \neq & \delta_{1}\neq \delta_{2}\neq \delta_{3}\neq \delta_{4}\neq \delta_{5}\neq \delta_{6}\neq \;0 \end{array}$$

The studied value of F-statistics can be used to accept or reject the null hypothesis. As stated by Ali et al. (54), long-term associations are present if the calculated F-statistical values are greater than the upper bounds value, no long-term association exists if the calculated F-statistical value is less than the lower bounds value, or if a calculated F-statistical value is between the upper and lower bound values.

### ARDL model

Chen et al. (55) proposed the Dynamic ARDL model. Compared to other time series models, the ARDL model has distinct advantages. Short temporal data can be used with the ARDL model, according to Dong (56). If the series is stationary at I (0), I(I), or both, the ARDL model can be used. Lags for independent and dependent variables can be used in various ways. A cointegration relationship exists between the variables studied, according to the results of ARDL bound tests. The long-term ARDL model looks like this:


(3) $$\begin{array}{l} LNFAi=\beta 0+\overset{q}{\underset{i=1}{\sum }}\delta 1\;LHEt-i\;+\overset{q}{\underset{i=1}{\sum }}\delta 2\;LGDPt-i\;\\ +\overset{q}{\underset{i=1}{\sum }}\delta 3\;LGCFt-i\;+\overset{q}{\underset{i=1}{\sum }}\delta 4\;LFDIt-i\;\\ \;+\overset{q}{\underset{i=1}{\sum }}\delta 5\;LICTt-i\;+\overset{q}{\underset{i=1}{\sum }}\delta 6\;LCEt-i\;+u_{\text{t}} \end{array}$$

The equation above refers to the long-term fluctuation in the study variables. Each variable's delays were chosen using the Akaike information criteria. The following error correction model was used for the short-run ARDL model:


(4) $$\begin{array}{l} LNFAi=\beta 0+\overset{q}{\underset{i=1}{\sum }}\beta 1\;\Delta LHEt-i+\overset{q}{\underset{i=1}{\sum }}\beta 2\;\Delta LGDPt-i\;\\ +\overset{q}{\underset{i=1}{\sum }}\beta 3\;\Delta LGCFt-i\;+\overset{q}{\underset{i=1}{\sum }}\beta 4\;\Delta LFDIt-i\;\\ +\overset{q}{\underset{i=1}{\sum }}\beta 5\;\Delta LICTt-i\;+\overset{q}{\underset{i=1}{\sum }}\beta 6\;VLCEt-i\;+\delta ECT_{\text{t}-1}+u_{\text{t}} \end{array}$$

A usual range for the error correction term is from 1 to 0 in the equation above, while represents the short-run variance and ECT represents the adjustment speed from disequilibrium. You need a negative and statistically significant error correction term to ensure any shock is absorbed in the next period. CUSUM and CUSMSQ were used to verify the model's stability (57). Breusch–Godfrey Lagrange Multiplier was used to verify serial correlation (LM). Breusch-Pagan-Godfrey (BG) and autoregressive conditional heteroscedasticity (ARCH) were employed to verify heteroscedasticity, whereas Jarque–Bera was used to verify residual normality. Using the Ramsey reset test to check the stability of the model.

### Dynamic autoregressive distributed lag simulations

Wang and Watson (58) introduced the dynamic ARDL model to research the short-run and long-run connection between the study variables to remove the difficulties of the existing ARDL. Using dynamic ARDL simulations, the graph of the regressor's change and the impact on the regress may be estimated, stimulated, and predicted automatically while the other variables in the equation remain constant. There must be cointegration between the variables being studied in order to apply the dynamic ARDL simulation method (59, 60). The vector of variables from the multivariate normal distribution was replicated 5,000 times using the dynamic ARDL error-correcting term technique. It is possible to see how the regressor changes over time and how that changes the regress. IEA and BERC (61) and Lin and Jia (62) used these terms to describe the ARDL bound error correction tests.


(5) $$\begin{array}{l} \Delta LNFA_{\text{t}}=\delta_{0}+\delta_{1}LNFA_{\text{t}-1}+\beta 1\Delta LHEt-i\\ +\delta_{2}LGDP_{\text{t}-1}+\beta 2\;LGDPt\;+\delta_{3}LGCF_{\text{t}-1}+\beta 3\;LGCFt\\ +\delta_{4}LFDI_{\text{t}-1}+\beta 4\;LFDIt\;+\delta_{5}LICT_{\text{t}-1}+\beta 5\;LICTi\\ +\delta_{6}LCE_{\text{t}-1}+\beta 6\;LCEt+u_{\text{t}} \end{array}$$

It is also important here to deliver the social importance of this study; therefore, this study stresses the role of training in promoting awareness and improving financial investment attitudes among the public. The role of financial assets could show a positive change in public health and may cause to increase in the level of human wellbeing in the long and short runs. In other words, China's economy's financial and health policies strongly impact the energy market. It means that increased spending on health initiatives produces more accuracy than other activities. Furthermore, improvements in health care facilities and the health of citizens can be a glimpse of transformed government policies toward the health sector with a rise in income in recent years. In other words, governments in the specified economy have employed more health-related strategies, leading to greater public investments in this sector. As a result, the positive changes in household social benefits may have a negative impact on the investment of financial assets in the long run. The results may show a significant association in the long and short ranges. However, household financial assets are expected to impact social wellbeing considerably. Put another way, households in the defined economy have both the incentive (from the health risk) and the self-assurance to participate in productive investment. True, in the post-pandemic era, an increase in household financial capability (ability and opportunity to act) might lessen the risk of investment in the health sector. This would have a major bearing on families' financial decisions. Therefore, governments can better increase or decrease their health spending shares by understanding the factors that affect the allocation and risk of financial assets in the case of China.

## Results and discussion

A normal distribution trend was confirmed by Jarque-Bera test statistics, with the average LNFA, LHE, LGDP, LGCF, LICT, LFDI, and LCE. Table 1 shows the results of the descriptive statistics. However, there is a no larger difference between each variable's mean and median values.

**Table 1:** Descriptive statistics test.

**Variable**	**LNFA**	**LHE**	**LGDP**	**LGCF**	**LICT**	**LFDI**	**LCE**
Mean	10.231	12.451	6.954	7.552	14.235	8.412	6.741
Median	10.114	12.234	6.852	6.987	14.125	8.222	6.552
Max.	12.521	15.423	8.456	9.456	20.658	12.563	9.562
Min.	8.245	6.559	3.854	3.854	6.458	5.446	5.458
Std. dev.	1.569	2.089	2.125	2.182	3.888	2.111	2.885
Skewness	−0.589	−0.564	−0.822	−0.556	−0.897	−0.4222	1.445
Kurtosis	2.09	2.46	3.85	2.11	3.31	2.12	5.845
J-B	3.56	3.12	5.23	2.85	1.844	3.852	1.558
Prob.	0.002	0.000	0.000	0.000	0.002	0.000	0.000

When utilizing statistical methods and approximation parameters, whether or not a series is stationary significantly impacts the empirical evidence. No unit root function in the equation means that data can increase or decrease over a set number of values for a long time. If that's true, then the data's constant variance must remain constant throughout time. In contrast, the likelihood function of non-stationary data exhibits state-dependent features in the stochastic technique. As previously stated, the long-term impacts of external shocks do not revert to equilibrium or the random walk condition. If a statistical model provides prediction partiality or pseudo-regression utilizing non-stationary data, the data's dependability must be checked (63–65). Many fundamental econometric theories and methodologies are based on the unit root test, determining if economic variables are stationary.

First, order one, I (1), must be established in the data series, notably the response variable, in order to examine the correlations among the dependent variables and their explanatory variables. Furthermore, no variables of order one or reflecting the seasonal unit roots (66–69) should be integrated. Phillip-Perron (PP) unit root and Augmented Dickey-Fuller (ADF) tests were utilized to inspect the variable's order to meet the qualifying requirements in this study. Table 2 shows the outcomes of the unit root tests used in the study. Under the null hypothesis of the unit root, PP and ADF are examined. As shown in Table 2, it is not possible to discard the null hypothesis of a unit root (PP and ADF) covering virtually all variables at the level I(O) but is rejected at first I (1) disagreement. Because of this, the co-integration of ARDL boundaries can be achieved by combining all data series in order one and level.

**Table 2:** ADF and PP unit root tests.

**Variable**	**ADF**		**PP**	
	**Level**	**Difference**	**Level**	**Difference**
LNFA	−0.8542	−3.6932^*^	−1.5527	−4.8841^*^
LHE	−1.2375	−7.4598^*^	−1.9952	−5.4458^*^
LGDP	−3.7465^*^	−5.6589	−3.8923^*^	−6.5245
LGCF	−1.2356	−4.8246^**^	−1.9994	−3.5655^**^
LICT	−3.2145^**^	−5.2258	−3.0896^**^	−6.8754
LFDI	0.2569	−7.5589^*^	1.2389	−9.4588^*^
LCE	−1.2251	−5.2369^**^	−2.8546	−6.2238^*^

*, ** 1% and 5% significance level.

Different lags can be selected for independent and dependent variables in an autoregressive distributed lag model. The lags selection criteria test results are shown in the following table. Selecting lags for independent and dependent variables is made more accessible by AIC, SC, and HQ. For lag selection, we employed the AIC, which found that the AIC value for lag two was the lowest of all three lag length selection criteria for our model. There are results for the lag selection criterion in Table 3.

**Table 3:** Lag selection criteria.

**Lag**	**AIC**	**SC**	**HQ**
0	−6.8852	−5.2333	−7.6691
1	−9.5524	−8.1155^*^	−10.524
2	−7.2569^*^	−9.8854	8.14897
3	−10.452	−13.4521	−17.5549^*^

* 1% and 5% significance level.

In order to investigate the long-term correlation between the research series, the ARDL bounds test was performed. F-statistics are shown in Table 4. Using F-statistics, the cointegration can be determined. At the 10, 5, and 2% significance levels, the estimated F-statistics value exceeds the upper boundaries, showing co-integration between the research variables.

**Table 4:** Bound test.

**Test**	**Value**	**K**
F-statistics	9.8844	5
**Critical bound value**		
**Significance**	**Lower bound**	**Upper bound**
1%	3.92	5.31
2.5%	3.41	4.71
5%	3.00	4.21
10%	2.57	3.69

The results of several diagnostic data are shown in Table 5. The model's consistency was examined using diagnostic statistics. Breusch-Godfrey LM test results show that the model utilized has no serial correlation problem. When the Breusch-Pagan-Godfrey and ARCH tests were used to look for any heteroscedasticity issues, the results showed none. The reset Ramsey test was used to determine if the model was accurately specified; findings show that the model is correct.

**Table 5:** Diagnostic tests.

**Diagnostic statistics test**	***X*** **^2^ (*P*-value)**	**Problem**
Breusch-Godfrey LM	0.666	N/A
Breusch-Pagan-Godfrey	0.899	N/A
ARCH	0.214	N/A
Ramsey RESET test	0.164	N/A

### Dynamic ARDL model outputs

**Figure 2:**
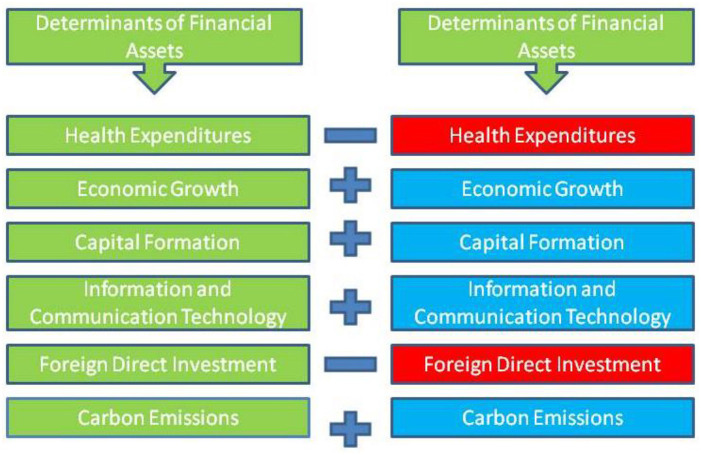
Graphical presentation of results.

Dynamic ARDL model outputs are summarized in Table 6. ARDL's originality lies in its capacity to reliably forecast the signs and number of changes in the factors and their short-term and long-term connectivity (Figure 2). For the classical ARDL technique, parameters' short- and long-term associations are of particular interest. According to the given coefficient of health expenditure, it is negatively associated with non-financial investment. This implies that a 1% rise in this factor would cause a reduction in investment by 0.451%. Health care expenses in China have risen steadily over time when total health care expenditures are considered. Health care in China is organized in a way distinct from other areas, including human resources, the economy, and strategic planning. China's healthcare system has unique challenges and opportunities compared to other emerging countries. In the recent decade, China's health sector has seen a dramatic increase in restrictions. Physical services, people resources, materials, technology, and healthcare services have undergone a major transformation. Two key portions guide this procedure. Healthcare services and social security are two of them. In particular, social security and healthcare have been separated, and a regulatory framework for healthcare has been established. Healthcare's Transition Program has played a critical role in this transformation. The impact of the healthcare transition program extends far beyond the last several decades. The progression of this procedure can be observed in the development plans. Health care's transition program has goals and solutions similar to development plans. Accordingly, the last two decades are critical to understanding how health care costs have evolved. Detailed information on cost-cutting strategies, improvements to be made, issues to be addressed, and tasks to be completed can be gleaned from development plans. Therefore, it needs time to overlook policies concerning health expenditures and investment in non-financial assets.

**Table 6:** Outcomes of dynamic ARDL.

**Variables**	**Coefficients**	**Std. error**	**T-statistics**	***p*** **-value**
Cons.	−0.5674^*^	0.2311	−2.4545	0.005
LHE	−0.4512	0.0991	4.5529	0.000
LGDP	0.7512	0.1356	5.5398	0.000
LCF	0.5562	0.3022	1.7412	0.002
LICT	0.8900	0.2545	3.4970	0.000
LFDI	−0.2345	0.0256	9.1601	0.005
LCO_2_	0.8562	0.4563	0.8763	0.000
ECT(-1)	−0.5678	0.1356	4.1873	0.000
R-Square	0.9256
*N*	29
F (*P*-value)	0.000^*^
Simulations	400

* 1% and 5% significance level.

Likewise, another determinant of non-financial investment is being considered economic progress. Economic growth is positively associated with the investment, which infers that a 1% rise in this factor would cause to increase in investment by 0.751%, respectively. From China's sample, GDP and growth are the essential factors of financial inclusion. This further suggests that the market size and growth are essential for attracting financial performance. This may be because high growth rates in a selected economy are frequently linked with high financial inclusion. As a result, China's rapid economic development has a favorable effect on GDP and NFA. Without a better expression, growth and NFA have become mutually reinforcing in the global economy. The link between NFA and economic growth has become increasingly endogenous as a result of globalization.

Likewise, the capital formation also plays a significant role in boosting financial investment. Therefore (70), according to the given coefficient of gross capital formation, it is positively associated with the explained variable, which infers that a 1% rise in this factor would cause to increase in non-financial investment by 0.556%. GCF's impact on NFA, applicable technical transfer, and economic growth, has been widely discussed. According to economic theory, gross capital formation affects the macroeconomic component directly and indirectly. Financial ramifications include the establishment of new businesses, the creation of jobs, and portfolio investments. Additionally (71), there is a direct gain from the transfer of technical knowledge, managerial skills, and other technological spillovers that encourage domestic demand for products and services, promoting capital accumulation. One aspect of economic growth that has drawn scholarly interest is the connection between GCF and financial inclusion. Gross capital formation is essential to business success and the economy's growth. However, it is a complicated procedure to create and strengthen capital formation. The asymmetric evaluation also shows a positive correlation between the GCF and NFA as a measure of GCF, and this correlation is statistically significant. In addition, linear correlations between NFA and GCF are impossible because they are modeled endogenously. Technology transfer leads to increased economic growth, which is the underlying assumption of this theory. Over time, it is projected that gross capital formation would boost financial inclusion, resulting in increased investment in financial assets.

The given coefficient value of FDI is positively associated with the non-financial investment, which infers that any significant change in foreign direct investment inflows would cause to decrease in the dependent variable by 0.234%, respectively. Domestic investment assesses how the domestic financial system affects FDI's contribution to economic growth. For example, if the investment variable is included and FDI or any financial development is considerable, this is read as FDI encouraging NFA through its efficiency level. As a result (72), it is not evident how FDI affects financial inclusion through investment or efficiency levels if this variable is not included. Countries with a well-developed financial system are better able to benefit from the advantages of FDI inflows; according to the study, financial inclusion and foreign direct investment (FDI) have a substantial correlation, according to the findings. China has a positive and statistically significant coefficient of FDI, but the coefficient of FDI at the 1% significance level is equally positive and significant. Even after controlling for financial development, this hypothesis holds, which shows that developing a solid domestic financial system may assist host countries in reaping the benefits of foreign direct investment more.

Also, information and communication technology (ICT) increases investment in the selected economy. Information and communication technology (ICT) has penetrated nearly every industry. The banking business, in particular, has seen radical transformations due to the widespread use of new technologies. A thriving banking sector signifies an economy's overall strength and resilience. The rise of the banking sector was greatly aided by ICT, allowing more people to participate in the economic process. By boosting back and administrative processes and front-end operations, ICT has enhanced banking efficiency while lowering client transaction costs, which has been the primary focus of ICT in financial management. Increasing numbers of banks and branches are implementing core banking systems, network computing, and customer relationship management using information and communications technology (ICT) (CRM). Using ICT, modern corporations may be more successful, have better governance, and provide value to the learning, activity, and management processes since it improves the company environment. Insight into the nature of technology and the influence of information and communication technologies on business and society is provided by ICT, which offers numerous perspectives. One of the modules of ICT in financial inclusion is required. Massive applications have resulted from the rapid growth and development of ICT in this sector. This article investigates how ICT applications and tools are employed in financial management. In addition (27), the paper explores how ICT tools and applications might be used to benefit a broader business audience. Workers' productivity skyrocketed as a result of their increased reliance on ICT. Workers' productivity is steadily rising as the banking business increasingly relies on technology.

Carbon emissions are also being used to investigate the behavior of the environment with non-financial investment, which shows the positive association between CO_2_ emissions and non-financial investments. The correlation between financial development and carbon emissions was statistically significant. Long-term carbon emissions climb by an average of 0.080 and 0.13% for every 1% increase in financial development. This study shows China's NFA levels have increased due to carbon emissions. A weaker financial system, more significant information asymmetry, and narrowing of financing channels through higher-cost capital lending help close the scale of products such as reduced employment, decreased rental equipment usage, and increased use of aging production lines that eventually show a positive trend in NFA and lead to carbon emissions. Improved servicing of consumption credit guarantees that consumers can acquire more items such as cars, appliances, and homes that contribute to the increase in carbon emissions due to a more developed financial system. In the same way, a rising stock market means a rising economy. Increased carbon emissions are caused by increased demand for energy due to increased output and consumption, which raises the confidence of consumers, businesses, and the economy. China's financial development has accelerated in the last four decades, contributing to an increase in carbon emissions (4). Increased access to external financing, in turn, aids in the expansion of investment, supporting the positive effect hypothesis, leading to an increase in carbon emissions due to China's growing financial development. In addition, China's stock market has a larger-scale impact on the country's economy and carbon emissions (73).

Error correction technique (ECT) is used to measure adjustment speed. At a 1% level of significance, the ECT value is negative and statistically significant. According to ECT, changes in the previous equilibrium occur at a rate of 0.56% per year. The R-squared number shows that the study's independent factors accurately reflect the dependent variable's 92% range of variation. The observed *P*-value of F-statistics indicates that the study's model is fitted correctly. Moreover, the smart chart for the estimated outcomes is given.

**Figure 3:**
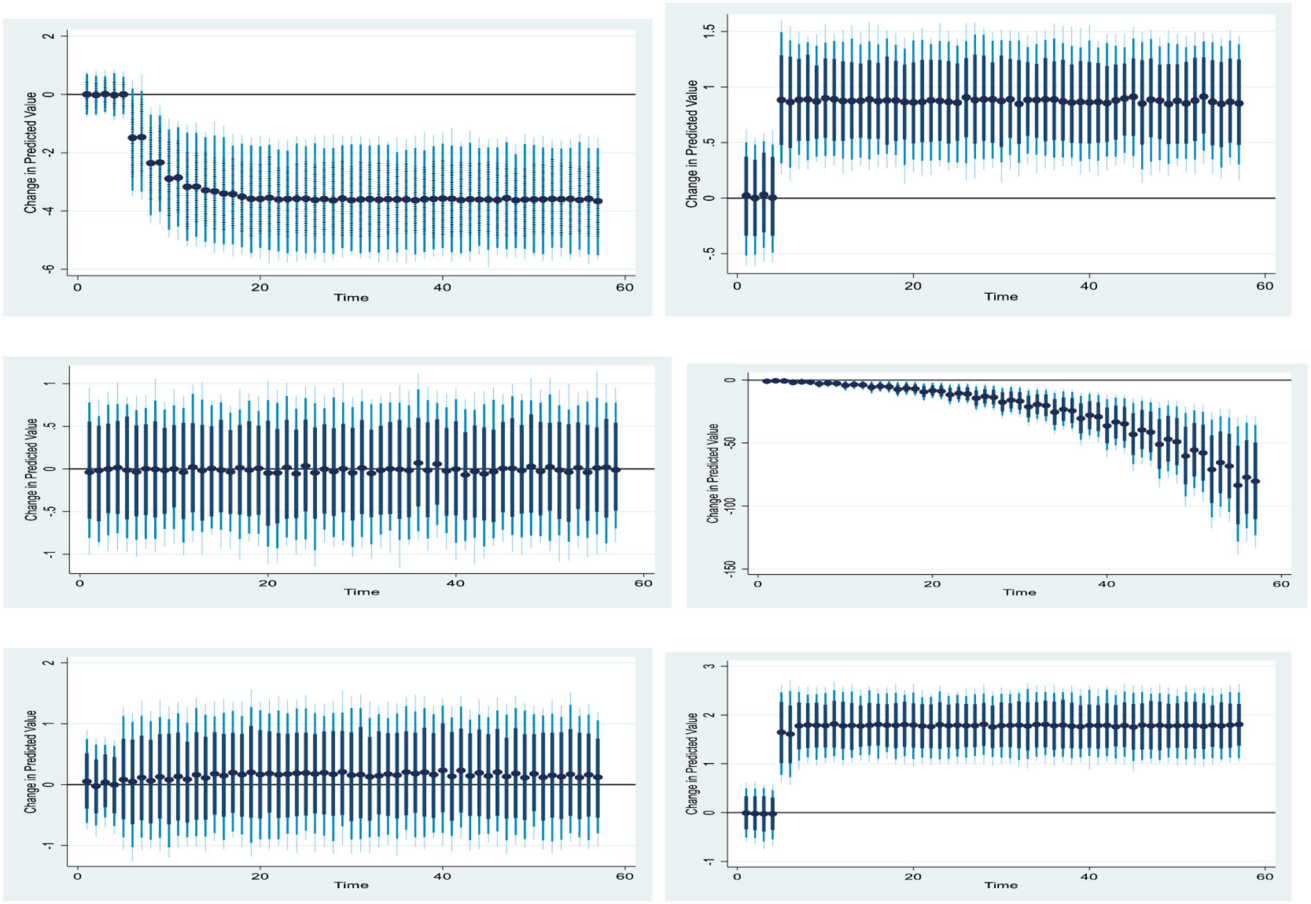
Graph for dynamic ARDL.

Figure 3 shows the 10% positive and negative shift in the pace of health expenditure and its effect on China's investment in non-financial assets. This shows the 10% positive change in China's negative health expenditures rate. Further, the trend for the graph can be seen in the diagram (Figure 3).

### Spectral causality test

**Figure 4:**
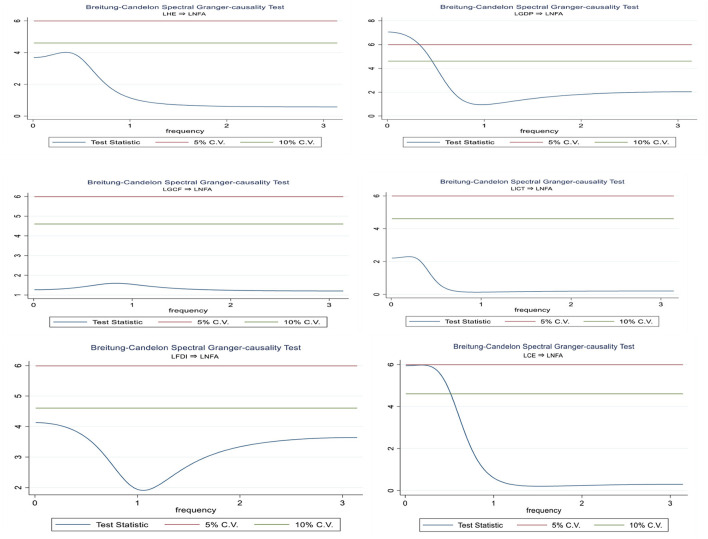
Graph for causality test.

Using Jia et al. (74) and Zhao et al. (75) frequency domain causality test after computing the long-run results, we can identify the causal relationship between the variables we've chosen to study. The null hypothesis of LNFA, LHE, LGDP, LGCF, LFDI, LICT, and LCE does not apply to Granger, as shown in Figure 4.

Before running the model, we employed pre-estimate approaches such as the Schwarz Bayesian information criterion, the sequential likelihood test and the Akaike information criterion, and Hannan and Quinn's information criterion to identify appropriate delays for the estimation. Bivariate models based on the frequency interval (0) yield the most important findings. Spectral Granger-causation results from the Breitung-Candelon spectral Granger-causality test show high unidirectional causality in all graphs except LFDI to LNFA.

## Conclusion and policy recommendations

This study looked at the impact of health expenditures on non-financial asset investment in China's economy from 1990 to 2019, taking insights from China's economy. Stochastic impact by a linear framework is the foundation of empirical modeling. Over the studied time in China, the ARDL-bound test indicated that these variables exhibit a long-run equilibrium relationship. To validate the validity of the results, Jordan and Philips used dynamic ARDL model simulations to predict long-run dynamics (2018). The findings of the simulations were intriguing. First, the estimates confirmed that health expenditures and foreign direct investment significantly decreased the impact on NFA in China. Second, the estimates show that economic growth, ICT, gross capital formation and carbon emissions significantly increase investment in NFA in the economy. Moreover, an advanced form of causality is also applied to investigate the causal association among variables.

### Policy recommendation

These empirical results provide important insights for policy decision-making toward achieving sustainable development in China. China has a strong government commitment to health care and is a global leader in this field. In addition, significant changes in how services are delivered are being mirrored by changes in how money is spent. Together with financial and service delivery regulations, they're enforced. Individuals' and society's health status, patient happiness and health institutions' financial discipline have all improved significantly due to the Transition Program in Healthcare. In addition to the above, China's health and financial inclusion will benefit greatly from implementing a comprehensive social security and health insurance programme. The efficient operation of the general health insurance and health coverage system is extremely important in recording and disciplining health expenditures and understanding the actual expenses.

Economic growth has different effects on the economy, depending on its nature. Increasing the economic progress makes increases NFA. In other words, economic growth can be counted as an injection complementary to investment. Therefore, an increase in economic growth in this economy leads to a boost in production and investment. A positive and significant long-run association between gross capital formation and NFA highlights the supportive role of gross capital formation on NFA. Interestingly, the impact of gross fixed capital formation on NFA is found to be positive. The positive significance of gross capital formation on NFA is in line with the investment-led growth in China's economy, which implies that the economy is struggling to catch up with investment.

Following this study's findings, the government and other stakeholders are strongly encouraged to improve the ICT capabilities of citizens in general, but this recommendation is especially directed at the poor. Many financial institutions are automating their services, which necessitates increased ICT capabilities. Using ICT platforms, financial service providers should make sure their services are user-friendly and written in plain English, according to the study's recommendations. That's why this study advises showing people the value of different digital content, which can motivate customers to expand their use of fundamental digital services, which will help spread the usage of digital into new areas. The young will be able to use ICT to obtain formal financial services and make informed decisions due to increased ICT capacity. Youth unemployment and poverty will decline as they can use formal financial services for investment. Citizens cannot take advantage of financial inclusion for their economic benefit if ICT levels do not rise, and they may remain impoverished for the rest of their lives.

It is not enough if a country has many investment opportunities, such as China. Most nations are now well-positioned to attract foreign investment thanks to technological advancements and an improvement in investment climates, which has increased the competition for FDI among numerous countries. If countries want to attract foreign direct investment (FDI), they must make conscious efforts. There is no doubt that China boasts a wide variety of investment options. In order to maximize the benefits of foreign direct investment, however, it is necessary to implement intentional strategies to attract, retain, and retain foreign direct investment. FDI will be promoted, facilitated, monitored, or regulated. Promotion of the country's investment opportunities will attract foreign investors; investment easing will confirm that the foreign investors are engaged and raise their investment wallets by radically decreasing the complications they combat in doing business in the state and investment management. Regulation will ensure that these foreign investments are not merely for the benefit of the investors but that they are also beneficial to the country as a whole. There has been a pressing need to revise environmental and financial inclusion policies in the previous few years.

It's important to note that there were a few problems with the data in this study. First, there was a notable omission from the data: people's current health conditions at home. Those who are ill but don't seek treatment and cannot afford medical care are automatically disqualified. This suggests that the current findings may be too conservative in assessing the effects of health shocks. Alternatively, a splurge on plastic surgery or a luxurious hospital room may be considered a luxury. That's why splurging on medical treatment can come out of your pocket. Our research assumes these events are relatively rare and that most unexpectedly high medical bills result from coverage for essential care rather than luxuries. This presumption is supported by a similar investigation which shows that health status influences catastrophic healthcare costs. It's also less probable that people may forgo essential purchases in favor of medical tourism. The second caveat was that it was hard to tell what caused what in terms of how household income was distributed and how much money people spent on health care. The timing of incidents was not captured in the available cross-sectional data. In our research, we found that low health care spending across all categories indicates households' employment of coping mechanisms to deal with the financial burden of unexpectedly high medical costs. We hypothesized that health problems emerged first, followed by household income and spending shifts.

## Data availability statement

The original contributions presented in the study are included in the article/supplementary materials, further inquiries can be directed to the corresponding author/s.

## Author contributions

All authors listed have made a substantial, direct, and intellectual contribution to the work and approved it for publication.

## Conflict of interest

The authors declare that the research was conducted in the absence of any commercial or financial relationships that could be construed as a potential conflict of interest.

## Publisher's note

All claims expressed in this article are solely those of the authors and do not necessarily represent those of their affiliated organizations, or those of the publisher, the editors and the reviewers. Any product that may be evaluated in this article, or claim that may be made by its manufacturer, is not guaranteed or endorsed by the publisher.
